# The effect of preoperative botulinum toxin a injection on traction force during hernia repair: a prospective, single-blind study, intra-patient comparison using contralateral side as a control

**DOI:** 10.1007/s10029-024-03087-9

**Published:** 2024-06-13

**Authors:** Soo Hyun Woo, Seok Joon Lee, Jun-Young Park, Eun Key Kim

**Affiliations:** 1 Department of Plastic Surgery, Chung-Ang University Hospital, Chung-Ang University College of Medicine, Seoul, Korea; 2 Department of Plastic Surgery, Asan Medical Center, University of Ulsan College of Medicine, 88 Olympic-ro 43gil, Songpa-gu, Seoul, 05505 Korea; 3 Department of Anesthesiology and Pain Medicine, Asan Medical Center, University of Ulsan College of Medicine, Seoul, Korea

**Keywords:** Botulinum toxins, type A, Hernia, ventral, Abdominal wall, Herniorrpaphy

## Abstract

**Purpose:**

Ventral hernias are a common complication of laparotomy, posing challenges particularly when primary fascial closure is unattainable. Although chemical component separation using preoperative botulinum toxin A (BTX) injections has emerged as a promising adjunct, objective evidence of its efficacy remains limited. This study aimed to objectively assess the effect of preoperative BTX on traction force during ventral hernia repair.

**Methods:**

A prospective, single-blind study was conducted on patients with midline incisional hernias following liver transplantation. BTX was administered unilaterally, and the traction force required to medially advance the anterior rectus sheath was measured intraoperatively. Pre- and post-injection CT scans were analyzed for changes in hernia size and LAW muscle measurements. Statistical analyses were performed to evaluate traction force differences between BTX-injected and uninjected sides.

**Results:**

Ten patients underwent hernia repair with primary fascial closure achieved in all cases. Comparison of pre- and post-injection CT scans showed no significant changes in hernia size. LAW muscle length increased by 1.8 cm, while thickness decreased by 0.2 cm. Intraoperative traction force measurements revealed a significant reduction on the BTX-injected side compared to the uninjected side (*p* < 0.0001). The traction force ratio on the BTX-injected to the uninjected side averaged 57%, indicating the efficacy of BTX in reducing tension.

**Conclusion:**

Preoperative BTX significantly reduces traction force during ventral hernia repair, highlighting its potential as an adjunctive therapy in complex cases. While challenges remain in patient selection and outcome assessment, BTX offers a promising avenue for enhancing abdominal wall reconstruction outcomes and reducing surgical complications.

## Introduction

Ventral hernias are one of the most common complications of abdominal surgery. Surgical options for repair of large hernias are often complex and limited, and tension-free primary fascial closure can be difficult in cases of loss of domain (LOD), which is known to increase the risk of wound complications and hernia recurrence. Despite advances in surgical technique and mesh technology, hernia recurrence rates after abdominal wall reconstruction (AWR) have been reported to be as high as 30% [1–3]. Several strategies have been proposed to improve the rate of fascial closure, including the component separation technique introduced by Ramirez in 1990, which has been shown to be effective in promoting primary fascial closure in large hernias. Technically, this involves medial advancement of the rectus abdominis muscle complex by external oblique release or transversus abdominis release [4].

However, these surgical techniques distort the anatomy of the abdominal wall and leave the possibility of subsequent functional insufficiency. In particular, open anterior component separation has been associated with complications such as skin necrosis and surgical site occurrences (SSO) related to the elevation of large skin flaps [5, 6]. Given the inherent problems of complex and extensive incisions, preoperative injection of botulinum toxin A (BTX) has shown promise as a chemical component separation. The clinical use of BTX in AWR was first described in 2009 when Ibbarra-Hurtado et al. showed that chemical paralysis of the lateral abdominal wall (LAW) increased abdominal wall compliance and lengthened the LAW muscles, allowing for successful primary fascial closure [7, 8].

Overall, the side effects of the chemical component separation in AWR are rare and transient and can be safely performed under image guidance, such as ultrasound [9]. However, the use of BTX for AWR is currently considered off-label and reimbursement is not always easy. Although existing clinical studies have consistently demonstrated the clinical benefits of chemical component separation, and several studies have compared imaging measures such as LAW muscle length and thickness or hernia defect size before and after BTX injection, few studies have provided objective evidence of the efficacy of preoperative BTX [8, 10–15]. The functional benefits of the procedure are even more difficult to demonstrate because incisional hernia is not a disease but essentially a surgical complication that is difficult to standardize due to the wide variation in systemic conditions, hernia size, abdominal wall compliance, or adhesions among patients. Furthermore, unlike surgical component separation, it is difficult to evaluate functional changes such as abdominal muscle compliance and medial advancement of the rectus abdominis complex before and after BTX injection.

Our group has previously demonstrated in animal models that preoperative BTX injections for AWR significantly altered defect size, traction force, postoperative intra-abdominal pressure, and recurrence rate in a rat model of elective hernia repair [16]. In the current study, we aimed to objectively determine the extent to which preoperative injection of BTX actually reduces the traction force to pull the anterior rectus sheath (fascia) medially during ventral hernia repair by comparing intraoperative tension on the injected and uninjected side in patients after unilateral BTX injection to determine the functional efficacy of the procedure.

## Materials and methods

This prospective, single-blind study enrolled patients aged 20 years and older presenting with midline incisional hernia following liver transplantation, which were referred to the Department of Plastic Surgery between March 2022 and February 2023. Exclusion criteria comprised individuals with connective tissue disease, skin defects, or enterocutaneous fistulae, as well as those with hypersensitivity to BTX and systemic neuromuscular junction disorders.

Hernia size inclusion criteria stipulated a maximum defect width exceeding 4 cm, classified as a ‘large ventral hernia’ according to the classification system proposed by European Hernia Society (EHS) members [17]. Additionally, the width was restricted to not exceed 8 cm to ensure the safety of fascial closure. This parameter was based on findings from a systematic review and meta-analysis by Timmer et al., which indicated that unilateral muscle lengthening achieved by BTX was approximately 3.2 cm, while hernia width reduction averaged approximately 3.5 cm [18].

Botulinum Toxin A (Botulax®, Hugel Inc., Seoul, Korea) was administered unilaterally by an anesthesiologist specialized in ultrasound-guided procedures. Randomization was employed to determine the side (left or right) of BTX injection. Both the administering anesthesiologist and the patient were aware of the injection site, while the operating surgeon remained blinded until traction force measurements were completed during hernia reconstruction surgery to uphold objectivity. BTX was prepared at a concentration of 150 units diluted in 72 cc of saline. This solution was divided into doses of 8 cc each, containing 16.6 units of BTX. Administration involved injection into three layers of the unilateral LAW muscles, specifically the external oblique, internal oblique, and transverse abdominis, at three distinct points, as described by Smoot and Elstner [19, 20].

Before BTX injection, patients underwent abdominal CT scans, with a follow-up scan approximately 4 weeks later, just before surgery. CT axial section views were utilized to measure the width of the fascial defect in each image, and the total fascial defect area was computed by integrating these widths with the section thickness. Comparative analyses were conducted between pre- and post-injection scans to evaluate changes in maximal fascial defect width, total fascial defect area, and LAW muscle length and thickness. Muscle length and thickness were measured from the axial image at the selected lumbar spine level (L3) as a reference point, with muscle thickness measured at its thickest point.

**Fig. 1:**
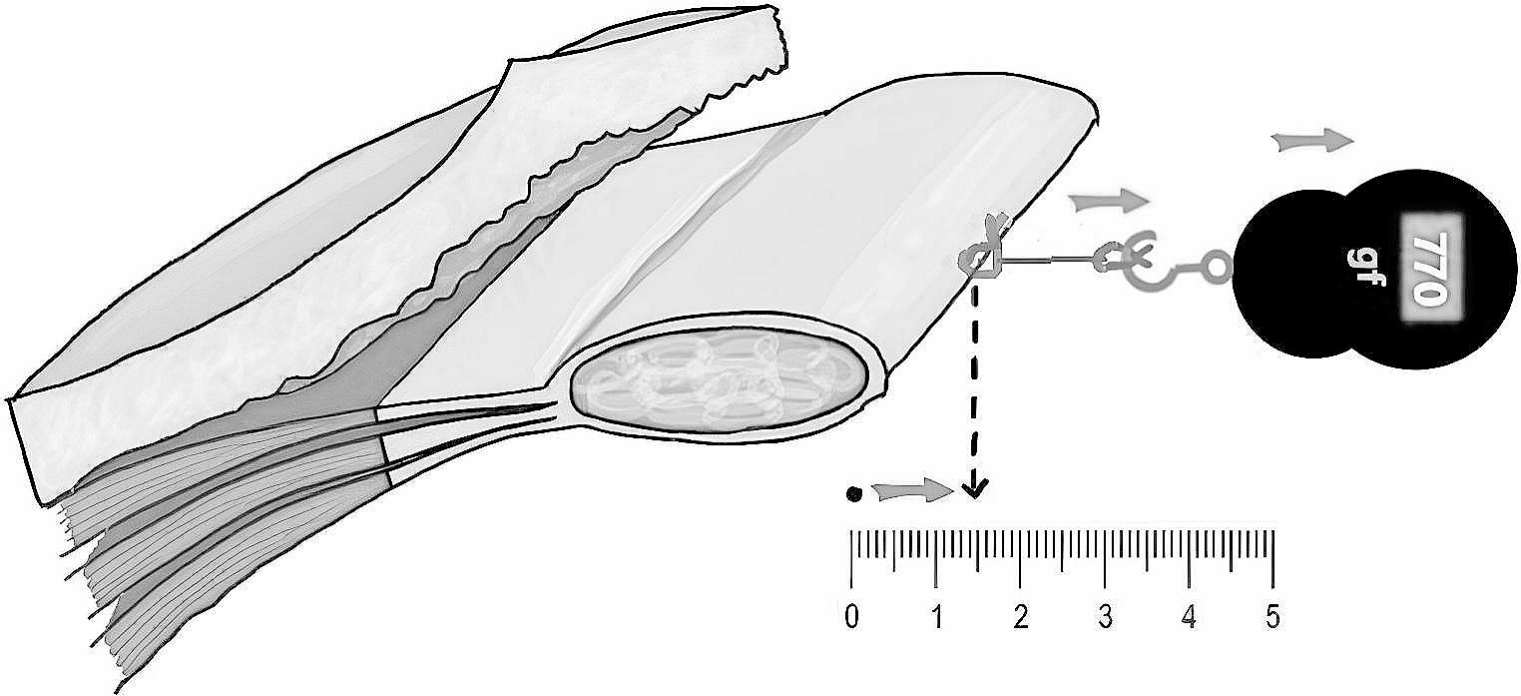
Traction force measurement The measurement was conducted using a digital scale, taken at 0.5 cm intervals from 1 cm to a maximum of 5 cm, or until the fascia was on the verge of tearing, even if beyond the midline. The example illustration shows a traction force of 770 gf measured at 1.5 cm

Four weeks following BTX injection, hernia repair was performed. Upon elevation of the skin flap to the linea semilunaris bilaterally following a midline incision, the traction force required for the medial advancement of the anterior rectus sheath at the maximum defect width level was measured. This measurement was conducted using a digital scale hooked to a 1 − 0 Vicryl loop sutured at the margin of the fascial defect, with a minimum of 1 cm bite. Measurements were taken at 0.5 cm intervals from 1 cm to a maximum of 5 cm, or until the fascia was on the verge of tearing, even if beyond the midline (Fig. 1). This process was carried out on both the BTX-injected side and the uninjected side of the abdomen. Unblinding of the operating surgeon occurred only after completion of the traction force measurements. Subsequent to unblinding, BTX was then injected into the previously uninjected side to maintain symmetry in postoperative muscle function. Hernia repair was finalized with myofascial coaptation, typically employing onlay mesh, unless refused by the patient or contraindicated. Participants were followed up 6 months post-discharge to assess hernia recurrence and complications.

Data analysis was performed using R version 4.3.2. The statistical significance of changes in maximal width of the fascial defect, total area of the fascial defect, and LAW muscle length and thickness was assessed using paired t-tests. To evaluate the impact of both size and group (BTX-injected vs. BTX-uninjected) on traction forces, a general linear model was employed. Additionally, the ratio of traction forces between the injected and uninjected sides (traction force of BTX-injected side/traction force of BTX-uninjected side) was analyzed using a general linear model. Repeated measurements were addressed using a covariance pattern model to accommodate the correlation between observations within the same patients.

This study received approval from the Institutional Review Board of the authors’ institution (2021 − 1242) and the Korean Ministry of Food and Drug Safety. It was conducted in accordance with the principles of the Declaration of Helsinki, with all patients providing voluntary agreement to participate after receiving informed consent. The consent form stated the off-label use of BTX and highlighted its limited known risks and side effects.

## Results

The study included a cohort of 10 patients who underwent hernia repair procedures between October 2022 and June 2023. Table 1 displays the demographics and preoperative characteristics of the patients. On average, the time interval between liver transplantation and hernia repair was 4.1 years (range: 1 to 11 years), with all patients maintained under immunosuppressive therapy.

**Table 1:** Patients’ demographics and preoperative characteristics

Characteristics		Mean ± SD (range)
Age		62.2 ± 3.71 (55–67)
Sex		
	Male	8
	Female	2
Body mass index		25.61 ± 4.91 (15.36–31.03)
Smoking		
	Yes	8
	No	2
Diabetes		
	Yes	5
	No	5
Liver transplantation		
	Cadaveric	1
	Living donor	9
Reason for LT (HCC)		
	Alcoholic LC	6 (3)
	HBV LC	2 (1)
	AIH LC	1 (0)
	Crpytogenic LC	1 (0)

LT: Liver transplantation; HCC: Hepatocellular carcinoma

All patients achieved primary fascial closure, with onlay mesh utilized in nine cases. Complications included one instance of an infected seroma and one case of partial necrosis of the wound margin. The average hospital stay duration was 7.7 ± 2.2 days. There were no reports of hernia recurrence during the six-month follow-up period.

Table 2 presents comparisons of measurements based on pre- and 4 weeks post-injection CT scans. The mean maximum defect width changed from 6.8 ± 3.3 cm pre-injection to 7.0 ± 3.1 cm post-injection, with no statistically significant difference observed (*p* = 0.665). Specifically, the width increased in five patients and decreased in five patients, resulting in a mean percentage change of + 6.91% ((post-injection width - pre-injection width)/pre-injection width). Similarly, the mean fascial defect area increased from 53.89 ± 47.53 cm² pre-injection to 59.85 ± 44.89 cm² post-injection, although this change was not statistically significant (*p* = 0.278). Notably, the area increased in six patients and decreased in four patients, resulting in a mean percantage change of + 21.0% ((post-injection area - pre-injection area)/pre-injection area). Regarding LAW muscle measurements, there was a tendency for muscle length to increase by 1.8 cm (*p* = 0.178), and a statistically significant decrease of 0.2 cm in muscle thickness (*p* = 0.009). Follow up CT scans > 6 months postoperation was available in 4 patients, which showed comparable LAW muscle length and thickness as pre-injection measurements (data not shown).

**Table 2:** Comparisons between pre- and post- BTX injection CT scan measurements

Measurement	Mean ± SD	*P* value
Fascial defect maximum width		0.665
pre-BTX, cm	6.8 ± 3.3	
post-BTX, cm	7.0 ± 3.1	
Fascial defect total area		0.278
pre-BTX, cm^2^	53.89 ± 47.53	
post-BTX, cm^2^	59.85 ± 44.89	
LAW muscle length (injected side)		0.178
pre-BTX, cm	21.0 ± 3.7	
post-BTX, cm	22.8 ± 5.1	
LAW muscle thickness (injected side)		0.009*
pre-BTX, cm	1.6 ± 0.3	
post-BTX, cm	1.4 ± 0.3	

BTX: botulinum toxin A, LAW: lateral abdominal wall

**Fig. 2:**
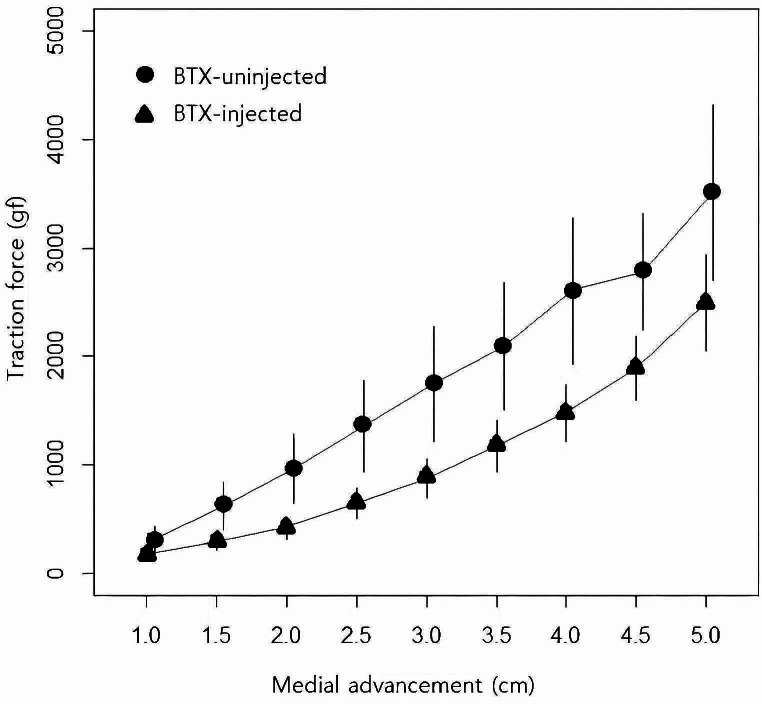
Mean traction force according to the distance of medial advancement The estimated mean traction force was 641.08 gf for BTX-injected group and 942.57 gf for BTX-uninjected group, indicating a significant difference (*p* < 0.0001). When the interaction effect between the two groups (BTX-injected and BTX-uninjected) regarding traction force (absolute value) across different distances were analyzed, the effect of BTX on traction force was significantly influenced by pulling distance (p for interaction = 0.0007)

The estimated mean traction force for the BTX-injected group was 641.08 gf with a 95% confidence interval ranging from 795.6 to 1089.55. In comparison, the BTX-uninjected group had an estimated mean traction force of 942.57 gf, with a 95% confidence interval spanning from 502.94 to 779.22, indicating a significant difference in traction force between the two groups (*p* < 0.0001, Fig. 2). For instance, at a distance of 1 cm, the traction force for the uninjected group measured 317.00 ± 174.98 gf, whereas for the injected group, it was 177.35 ± 76.79 gf. As the distance of medial advancement increased to 3 cm, these values escalated to 1748.60 ± 744.52 gf for the uninjected group and 879.80 ± 262.72 gf for the injected group. By the time the distance reached 5 cm, the traction force for the uninjected group was 3509.00 ± 774.95 gf, while for the injected group, it measured 2498.33 ± 581.86 gf. Notably, four patients experienced fascial tears beyond the 4 cm mark, rendering complete measurement unattainable for them. When the interaction effect between the two groups (BTX-injected and BTX-uninjected) regarding traction force (absolute value) across different distances were analyzed, there was a significant interaction between the group and distance factors on traction force (p for interaction = 0.0007), or the effect of BTX on traction force was significantly influenced by pulling distance.

**Fig. 3:**
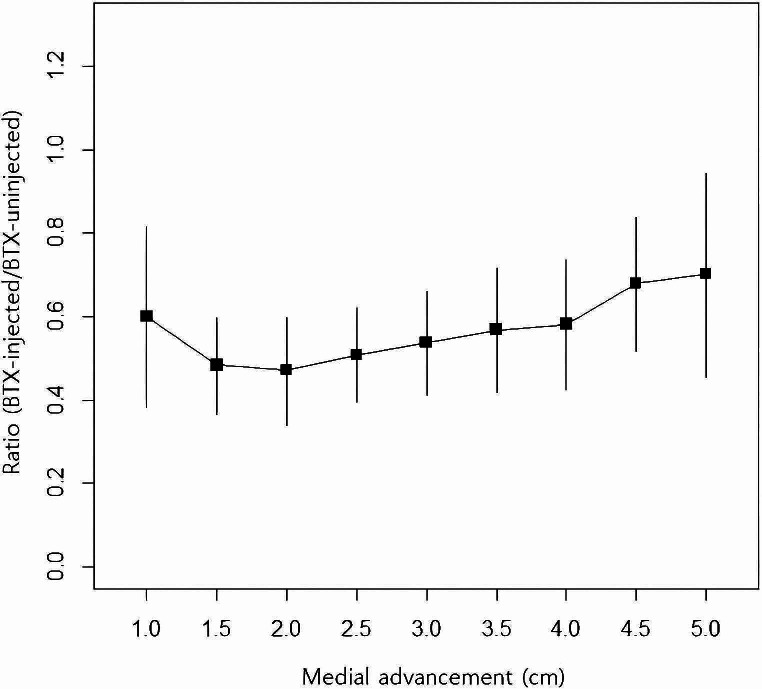
Traction force ratio comparing the injected side to the uninjected side across distances of medial advancement BTX reduced the traction force required for the medial advancement of the anterior rectus sheath to 57% on average. There was a trend toward a slight increase in the ratio on the injected to the uninjected side from 2 cm onward, without statistical significance (*p* = 0.783)

Upon analyzing the traction force ratio on the BTX-injected to the BTX-uninjected side, it was observed that BTX reduced the traction force required for the medial advancement of the anterior rectus sheath to 57% on average, as illustrated in Table 3; Fig. 3. There was a trend toward a slight increase in the ratio on the injected to the uninjected side from 2 cm onward, without statistical significance (*p* = 0.783).

**Table 3:** Traction force ratio comparing the BTX-injected side to the uninjected side

Distance (cm)	Number	Ratio (mean ± SD)	95% CI
1	10	0.60 ± 0.31	0.38–0.82
1.5	10	0.48 ± 0.16	0.37–0.60
2	10	0.47 ± 0.18	0.34–0.60
2.5	10	0.51 ± 0.16	0.40–0.62
3	10	0.54 ± 0.18	0.41–0.66
3.5	9	0.57 ± 0.20	0.42–0.72
4	9	0.58 ± 0.20	0.43–0.74
4.5	8	0.68 ± 0.19	0.52–0.84
5	6	0.70 ± 0.23	0.46–0.95

## Discussion

In cases of complex ventral hernias where primary fascial closure is unattainable, preoperative BTX injections have emerged as an adjunct along with preoperative progressive pneumoperitoneum (PPP), and these can also be combined with conventional surgical component separation methods. Systematic reviews have evaluated their efficacy and safety, yet the current evidence remains limited due to small sample sizes, study heterogeneity, and variations in BTX administration protocols [2, 21]. Despite these limitations, the clinical utility of chemical component separation using BTX in complex abdominal wall reconstruction (AWR) has been consistently recognized. Studies by Bueno-Lledó et al. and Deeredberg et al. have reported favorable outcomes with preoperative BTX, demonstrating successful downstaging of large ventral hernia reconstructions and higher rates of primary fascial closure, lower wound infection rates, and comparable recurrence rates through prospective comparison and propensity-scored matched study, respectively [14, 22]. Marturano et al. found BTX to yield outcomes similar to surgical component separation while reducing the incidence of surgical site occurrences [15]. However, establishing an appropriate control group in clinical studies to demonstrate the effectiveness of BTX remains challenging due to the different types of ventral hernias, the diverse techniques used for hernia repair and the use of heterogeneous meshes for durable repairs. In addition, although CT scans are helpful in preoperative planning, their utility in assessing abdominal wall compliance or intra-abdominal adhesions is limited [23].

Initial efforts to objectively demonstrate the benefit of BTX prior to AWR were based primarily on anatomical measurements before and after injection, primarily by measuring the length and thickness of the LAW muscle and the size or width of the fascial defect. Changes in LAW muscle length and thickness have been largely consistent, with BTX increasing length and decreasing thickness [8, 12, 18, 24]. Some studies have reported that BTX significantly reduces the width of hernias. However, in a meta-analysis, the study results showed high heterogeneity [7, 8, 18]. In 2023, Claessen et al. [13] reported that computed tomography studies showed that BTX increased the transverse diameter and decreased the anteroposterior diameter in the abdominal cavity. Furthermore, although the median hernia width was significantly reduced, the hernia width actually increased in almost half of the patients, and the change in hernia width was influenced by the initial hernia width. In addition, interpatient heterogeneity was also found for dimensions such as abdominal width, depth and LOD, suggesting that they may be inadequate as objective indicators. In our study, the maximum width and area of the hernia did not change statistically significantly and actually increased in half of the patients. In addition, although the results of Claessen et al. were consistent with the literature for changes in length and thickness of the LAW muscle, the magnitude of the changes were smaller than those reported in previous clinical studies, which the authors attributed to the fact that previous studies generally enrolled patients with more severe defects with larger LODs, which would be expected to be difficult to reconstruct using conventional methods [13]. This is similar to our results, where we found that unilateral BTX injections in ventral hernia patients with defect widths between 4 and 8 cm resulted in an average increase in ipsilateral LAW muscle length of 1.8 cm and a decrease in thickness of 0.2 cm, with notably smaller changes than in previous studies. These findings highlight the difficulty in objectively demonstrating the magnitude of benefit from BTX, as well as the challenges associated with appropriately selecting patients who will actually benefit from BTX pretreatment.

In hernia repair, tension has often been recognized as an important intraoperative indicator [25]. While tension in the intact abdominal wall can be calculated using La Place’s law, tension in hernia repair is generally described as the pulling force transmitted axially. In our measurements, we used the term “traction force”, which follows a similar concept and measurement technique to “tension” in previous studies. Dragu et al. proposed intraoperative tensiometry as a decision-making tool as early as 2009 [26], and existing clinical studies investigating the effect of component separation on tension reduction have measured the effect of each stage of surgical release. Afifi et al. found that open anterior component separation resulted in approximately 70% reduction in tension [27]. Parikh et al. observed a reduction in tension from 7.4 lbs to 2.7 lbs with anterior release and 7.56 lbs to 4.35 lbs with posterior component separation with transversus abdominis release (TAR) [28].

We hypothesized that it would be appropriate to measure the change in traction force required for specific distances of medial advancement to evaluate the efficacy of preoperative BTX. The study standardized the patient population to patients with 4–8 cm wide median ventral hernias following liver transplantation and receiving immunosuppressive medications according to a uniform procedure and regimen at a single center. Unlike surgical component separation, there is no real way to measure traction force before BTX injection, so we injected BTX unilaterally and directly compared the traction force required to pull the rectus sheath medially on the injected side and the contralateral side using a digital scale. Because our study compared traction forces over a fixed distance on each side, the effect of hernia size on the results would be minimal. We found that the difference in absolute force between the BTX-injected and uninjected side was significant for medial advancement from 1 cm to 5 cm, with a significant interaction between group (BTX-injected versus BTX-uninjected) and distance on traction force. However, when we analyzed the ratio of the two groups’ traction forces, BTX reduced the tension required for a given amount of medial advancement to an average of 57% compared to the contralateral side, and this ratio was consistent across all distance intervals. However, the effect of BTX on tension tended to decrease with increasing distance, especially at distances greater than 4 cm, which may be due to the smaller sample size or partially due to the effect of static tension in the soft tissues. This reduction in tension with BTX pretreatment is not directly comparable, but appears to be somewhat less than the effect of surgical component separation in previous studies.

Our study has several limitations. First, our study was conducted in post-liver transplant patients, and all patients were taking immunosuppressive medications, which may require caution in the generalization. Although we randomized the sides, the muscles and adhesions on both sides may not be completely symmetrical due to liver transplantation techniques. In addition, the hernia size was relatively small compared to previous BTX studies and traction force (tension) was only measured at one point of maximum hernia width. As Tenzel et al. point out, despite its importance, the methodology for measuring tension is imperfect and less standardized [25], making it difficult to compare our results with previous studies, and its interpretation and utilization in clinical practice remains a challenge, as Hope et al. reported no correlation between hernia defect width and abdominal wall tension measurements [29].

The variety of preoperative assessment tools would influence the surgeon’s intraoperative decision making and preoperative planning. Ideally, appropriate preoperative assessment would exclude patients who would achieve primary fascial closure without BTX and include those who would avoid surgical component separation, downstage surgical repair, or avoid bridge mesh repair with BTX and/or PPP. On the other hand, Espinosa-de-Los-Monteros et al. reported that in patients undergoing hernia repair with TAR for complex abdominal wall defects, preoperative adjunctive BTX had no significant effect on postoperative intra-abdominal pressure or pulmonary plateau pressure [30]. This suggests that further research is needed to investigate the additional benefit of combining specific surgical procedures with chemical paralysis of the LAW muscles. Finally, although the indication for BTX in clinical practice would be realistically judged by the width of the defect, and the efficacy should be demonstrated by the outcome of increased primary fascial closure rate and decreased hernia recurrence rate through large clinical trials, it is necessary to design a high quality study with functional and dynamic variables such as abdominal wall compliance, closing tension, or intra-abdominal pressure.

In conclusion, our study adds to the growing body of evidence supporting the use of preoperative botulinum toxin A (BTX) injections as an adjunctive treatment for complex ventral hernias when primary fascial closure is not feasible. Despite the limitations and challenges outlined in the Discussion, our results demonstrate a significant reduction in traction force by an average of 43%, indicating the efficacy of BTX in facilitating medial advancement of the anterior rectus sheath during hernia repair. While further research is needed to address the complexities associated with patient selection, measurement techniques, and the comparative effectiveness of BTX versus other surgical approaches, our study underscores the potential of BTX as a valuable addition to the abdominal wall reconstruction armamentarium. Optimizing the management of complex ventral hernias requires a multidisciplinary approach that integrates both surgical expertise and innovative adjunctive therapies such as BTX to improve patient outcomes and reduce hernia recurrence as well as surgical complications.

## Data Availability

The datasets used and/or analyzed during the current study are available from the corresponding author on reasonable request.
